# Effects of combined radiotherapy with immune checkpoint blockade on immunological memory in luminal-like subtype murine bladder cancer model

**DOI:** 10.1080/15384047.2024.2365452

**Published:** 2024-06-11

**Authors:** JiaMin Huang, Eva Michaud, Surashri Shinde-Jadhav, Sabina Fehric, Gautier Marcq, Jose Joao Mansure, Fabio Cury, Fadi Brimo, Ciriaco A. Piccirillo, Wassim Kassouf

**Affiliations:** a Cancer Research Program, Research Institute of McGill University Health Center, Montréal, QC, Canada; b Division of Urology, Department of Surgery, McGill University Health Center, Montréal, QC, Canada; c Department of Radiation Oncology, McGill University Health Center, Montréal, QC, Canada; d Department of Pathology, McGill University Health Center, Montréal, QC, Canada; e Department of Microbiology and Immunology, McGill University, Montréal, QC, Canada; f Infectious Diseases and Immunology in Global Health Program, The Research Institute of the McGill University Health Centre (RI-MUHC), Montréal, QC, Canada; g Centre of Excellence in Translational Immunology, Montréal, QC, Canada

**Keywords:** Bladder cancer, radiotherapy, immunotherapy, combination therapy, immune checkpoint inhibitors, immune memory

## Abstract

MIBC is a highly lethal disease, and the patient survival rate has not improved significantly over the last decades. UPPL is a cell line that can be used to recapitulate the luminal-like molecular subtype of bladder cancer and to discover effective treatments to be translated in patients. Here, we investigate the effects of combinational treatments of radiotherapy and immunotherapy in this recently characterized UPPL tumor-bearing mice. We first characterized the baseline tumor microenvironment and the effect of radiation, anti-PD-L1, and combinatorial treatments. Then, the mice were re-challenged with a second tumor (rechallenged tumor) in the contralateral flank of the first tumor to assess the immunological memory. Radiation slowed down the tumor growth. All treatments also decreased the neutrophil population and increased the T cell population. Anti-PD-L1 therapy was not able to synergize with radiation to further delay tumor growth. Furthermore, none of the treatments were able to generate immune memory. The treatments were not sufficient to induce a significant and lasting pool of memory cells. We show here that anti-PD-L1 treatment added to radiotherapy was not enough to achieve T cell-mediated memory in UPPL tumors. Stronger T cell activation signals may be required to enhance radiation efficacy in luminal-like bladder cancer.

## Introduction

Bladder cancer is the fourth most common cancer in men and the 8^th^ leading cause of cancer death in the United States. It is estimated that there will be more than 81,180 new bladder cancer cases in 2022.^1^ Approximately 30% of all bladder cancer cases are muscle-invasive (MIBC), a lethal disease with a 5-year survival rate of less than 50%.^2^ The management of MIBC includes the standard treatment of radical cystectomy with neoadjuvant chemotherapy and radical cystectomy, where the patient’s bladder and surrounding organs are removed. Radiation-based therapy is the most widely utilized form of bladder-preserving strategy for MIBC.^3^ After 3 decades, progress is still slow in the treatment of MIBC, despite the aggressive multimodal treatment of neoadjuvant chemotherapy followed by radical cystectomy.^4^ Additionally, 40% of the patients are unfit to undergo surgery. Furthermore, nearly half of the patients presenting with MIBC will develop metastasis, leading to the 5-year survival rate dropping to 15%.^5,6^

Bladder cancer can be classified into different molecular subtypes, each with its own gene signatures and characteristics. For MIBC, the two main molecular subtypes are basal and luminal.^7^ Different subtypes have been associated with responding to different treatments and may be associated with different clinical outcomes.^8,9^ Luminal-like bladder cancer cell lines have been found to be more sensitive to radiotherapy compared to basal-like cell lines.^10–12^ There are few syngeneic-murine bladder cancer cell lines generated from the common mouse strain, and even fewer representing the luminal molecular subtype.^13^ The recently generated UPPL cell line is the only murine syngeneic bladder cancer cell line that has been found to recapitulate human luminal bladder tumors and could be used to assess treatment effects in murine models.^11^ Compared with the basal counterpart, the luminal molecular subtype has been found to have lower immune cell infiltration.^11^

In this study, we sought to understand the effects of combinational therapy of radiation and ICI in the luminal-like molecular subtype. Radiotherapy (RT) is an alternative treatment that allows bladder preservation and maintains the quality of life. RT can enhance tumor antigen release and presentation, but it can also increase the expression of programmed-cell-death1(PD-1) on T cells and programmed-cell-death-ligand1 (PD-L1) on cancer cells,^14^ likely in response to increased T cell activation. The immune suppression mediated by PD-1/PD-L1 may be prevented by the addition of anti-PD-L1 molecules. By using radiotherapy to enhance antigen presentation coupled with immunotherapy to prevent immune exhaustion, we may increase global T cell responses and ensure anti-tumor immunity. The establishment of immunological memory is also an important aspect for more sustained prevention of recurrence and metachronous metastasis. Indeed, almost 75% of high-risk bladder cancer patients will experience recurrence, leading to death.^15^ Enhanced antigen recognition and ensuing T cell activation and expansion are key to the generation of memory; thus, combining treatment modalities using RT and immune checkpoint inhibition (ICI) could promote and sustain immune memory toward tumors.^16,17^ Enhancing tumor antigen presentation and tumor antigen-specific T cell activation is also key to turning cold tumors into hot tumors which are more sensitive to treatments such as immunotherapy, currently being investigated in combination with radiation therapy.^18,19^ There are also ongoing clinical trials testing whether the combination of ICI with radiation and chemotherapy has better responses than those treatments without immune checkpoint therapy.^20,21^ These combinatorial treatments would activate the essential signals, namely antigen recognition, co-stimulation, and cytokine-mediated differentiation and expansion, all required for optimal T cell activation which are needed for the development of T cell memory.^19,22–24^ Thus, the goal of the present study is to assess whether a combination of anti-PD-L1 treatment and radiotherapy can potentiate immunological memory in immune-cold luminal-like UPPL tumors to prevent recurrence *in vivo*. We demonstrate that the UPPL mouse model is a good representative of luminal-like human tumors. We also show that RT and ICI combined were not sufficient to restore immune infiltration and immune memory to the TME, suggesting intrinsic immune-resistant mechanisms to this tumor that have yet to be overcome.

## Results

### UPPL tumors are characterized by reduced infiltration of lymphocytes and high neutrophils

**Figure 1.**
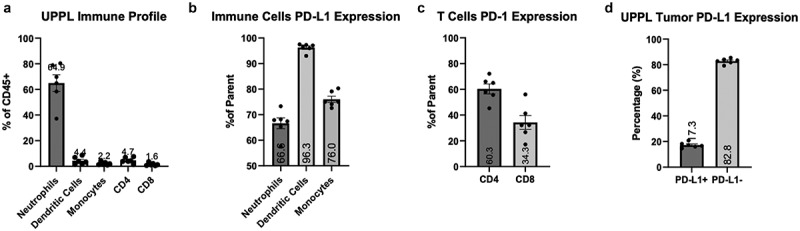
Immune profile of UPPL tumors and PD-L1/PD-1 expression within the tumor micro-environment. A. Percent of CD45^+^ of neutrophils, dendritic cells, monocytes, CD4+ T cells and CD8+ T cells (n=6) B. Percentage of cells expressing PD-L1 within their respective populations (neutrophils, dendritic cells, and monocytes). C. Percentage of cells expressing PD-L1 within CD4+ T cells and CD8+ T cells compartments. D. percentage of tumor cells (gated on CD45^−^ cells) expressing PD-L1. Number of columns indicate mean frequency.

UPPL tumors were recently generated using *Upk3a-Cre*^*ERT2*^; *Trp53*^*L/L*^; *Pten*^*L/L*^; *Rosa26*^*LSL-Luc*^ mice, and tumors recapitulate the luminal molecular subtype of bladder cancer with papillary histology and lower levels of immune infiltration in comparison with basal tumors.^11^ Hence, to confirm the immunologically cold status of UPPL tumors, we first examined immune infiltration in primary untreated tumors at their endpoints (1.5 cm^3^). We evaluated the immune cell composition in the tumor microenvironment (TME) by multi-parametric flow cytometry following excision by flow cytometry and assessed levels of PD-1/PD-L1 expression on tumor cells and antigen-presenting cells (APCs). Within the immune cell compartment (CD45^+^), neutrophils were the most abundant cells with a median of 64.9% of the CD45-positive fraction (Figure 1a). Additionally, we observed low levels of infiltrating T cells (4.6% CD4^+^ T cells and 1.6% CD8^+^ T cells) (Figure 1a) and noted a high CD4^+^/CD8^+^ T cell ratio. Moreover, we found high levels of PD-L1 in all three APC populations included in the analysis: neutrophils, monocytes, and dendritic cells (Figure 1b). The latter presented the highest frequency of PD-L1-expressing cells (96.3%) compared to neutrophils and monocytes (Figure 1b). In T cells, we noticed that CD4^+^ T cells trended toward a higher frequency of PD-1 positivity than CD8^+^ T cells (Figure 1c), which could imply that CD4^+^ T cells might be more exhausted than the CD8^+^ T cells. PD-L1 expression in tumor cells, gated as live and non-CD45^+^ cells, remained modest, consistent with luminal tumors (Figure 1d). Overall, the high frequencies of PD-L1-expressing cells among APC populations and the lower expression of PD-L1 on tumor cells suggest that the anti-PD-L1 blockade treatment could preferentially affect the immune compartment rather than tumor cells within this experimental cold tumor model.

### Radiation therapy slows UPPL tumor growth and alters the immune landscape of the TME

**Figure 2.**
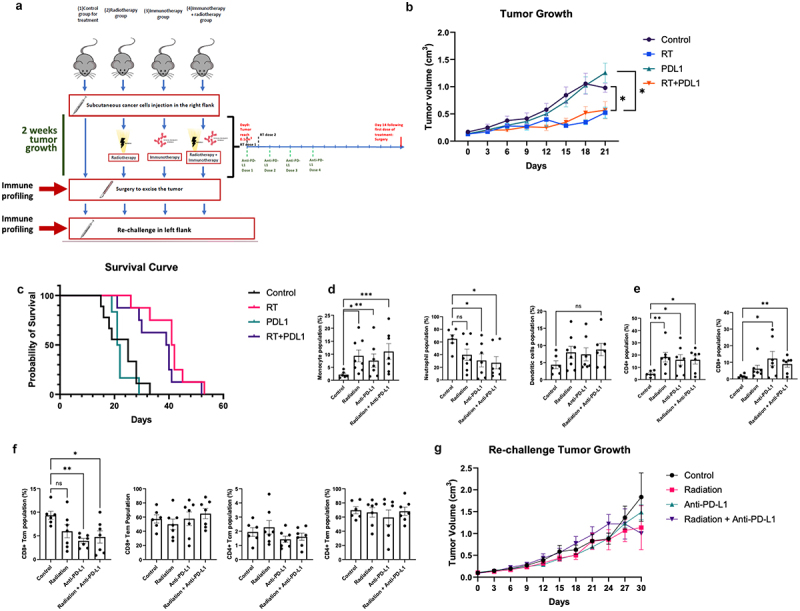
Radiation slows tumor growth, modifies the TME and does not impact re-challenge. A. Schematic of the treatment plane for each group (n=6-8). B. Tumor growth upon tumor reaching the size of 0.1cm^3^ for each different treatment arm. C. Kaplan-Meier survival curve analysis for each treatment group starting from the day the reaching the size of 0.1cm^3^. D. Monocytes, neutrophils, and dendritic cells frequencies (gated on CD45^+^cells) in the TIME following two weeks of treatment. E. CD4+ and CD8+ T cells frequencies in the TME following two weeks of treatment. F. Primary tumor TME effort and central memory T cells frequencies in the TME. G. Re-challenge tumor growth, on the opposite flank of the primary tumor upon reaching 0.1cm^3^ for each different treatment group.*= *P* < 0.05; **= *p* < 0.01.

Having established baseline immune infiltration patterns in the model, we next sought to evaluate the effect of radiation and/or anti-PD-L1 treatment on UPPL biology. We have previously shown that radiation and anti-PD-L1 combination therapy can synergize to slow tumor growth in the basal-like MB49 model, which is considered a ‘hot’ tumor.^25^ In the current study, we wanted to investigate whether it would have the same impact on UPPL tumors. Following the subcutaneous injection of UPPL cells into the right flank of the mice, we followed the tumor growth until it reached a palpable and measurable size of 0.1 cm^3^. Once reached, the mice were randomized into four different treatment groups and treated accordingly: Control, RT, Anti-PD-L1 and RT + Anti-PD-L1 (Figure 2a). We observed that RT significantly slowed the tumor growth in the 3 weeks following treatment compared to the control group and was associated with longer survival (*p* ≤ .05) (Figure 2b,c). Interestingly, treatment with anti-PD-L1 alone did not slow tumor growth (Figure 2b). More importantly, anti-PD-L1 treatment did not synergize with the effect of radiation in the combination arm, as both tumor growth and survival curve did not differ significantly from those of the RT arm (Figure 2b,c). The lack of effect of ICI on tumor growth in this model recapitulates previous findings on the effects of anti-PD-1 therapy in UPPL tumors.^11^

Knowing that UPPL is sensitive to RT, we were then interested in the immune-potentiating effects of both RT and ICI in the UPPL TME. Thus, we evaluated the immune cell composition in the TME by multi-parametric flow cytometry of each arm after 2 weeks of their respective treatments. Tumors from all treatment arms presented significantly higher frequencies of monocytes compared to untreated counterparts (Figure 2d). Interestingly, we observed a significant decrease in neutrophil frequencies in groups treated with anti-PD-L1 (Figure 2d), while populations of dendritic cells appeared unaffected by any treatment (Figure 2d). All treatments increased the frequencies of CD4^+^ T cells within the TME, while only the anti-PD-L1 treatment groups led to significant increases in CD8^+^ T cells frequencies (Figure 2e). Overall, we see an increase in monocytes and T cells and a decrease in neutrophils due to the treatments. This suggests that PD-L1 might promote neutrophil recruitment in the UPPL TME and may be detrimental to potent anti-tumor immune responses.

### Immunological memory is not affected by the combined RT and ICI

RT and PD-L1 blockade led to observable changes in the composition of both the myeloid and the T cell compartments, which could potentiate the conversion from cold to hot tumors. Particularly, the influx of T cells following RT and/or ICI treatment may be leveraged for subsequent therapies if they can induce long-lasting immunity. Immune memory of previously encountered tumor antigens may indeed help prevent tumor dissemination and recurrence at the primary site. T cell memory can be classified into effector memory (T_EM_) and central memory (T_CM_).^26,27^ T_EM_ tends to be more present in peripheral tissues and can respond rapidly, while T_CM_ tends to be found in lymphoid organs and has a better capacity to be proliferative.^28,29^ CD8^+^ or CD4^+^ T_EM_ can be defined as CD44^+^CD62L^−^ and CD8^+^ or CD4^+^ T_CM_ as CD44^+^CD62L^+.26,27^ CD62L is a marker to distinguish central memory from effector memory as CD62L controls the traffic to and from lymphoid organs.^30^

Following the excision of the primary tumor, we then assessed the baseline memory cells within the TME. In the primary tumor TME, we did not find any difference in either T_EM_ or T_CM_ subsets after two weeks of treatment, except an overall decrease in CD8^+^ T_CM_ in all treatment groups compared to control (Figure 2f). This suggests that while RT and ICI can increase T cell influx to the TME, the memory generation of these T cells may still be impaired by a potentially highly suppressive TME.

Hence, we next interrogated the effect of our combinational approach on immune recall responses upon re-exposure to the tumor. To experimentally mimic recurrence, primary UPPL tumors were excised surgically after 2 weeks of treatment and mice were re-challenged with a new UPPL tumor on the opposite flank (Figure 2a). Previous treatment to the primary tumor did not impact either tumor cell engraftment or the re-challenged tumor growth (Figure 2g). This secondary tumor growth was also not significantly different from the primary tumor growth (data not shown). Consequently, re-challenging these mice leads to unchecked tumor growth, similar to the primary tumors.

### The immune cell composition of rechallenged tumors is different from primary tumors

**Figure 3.**
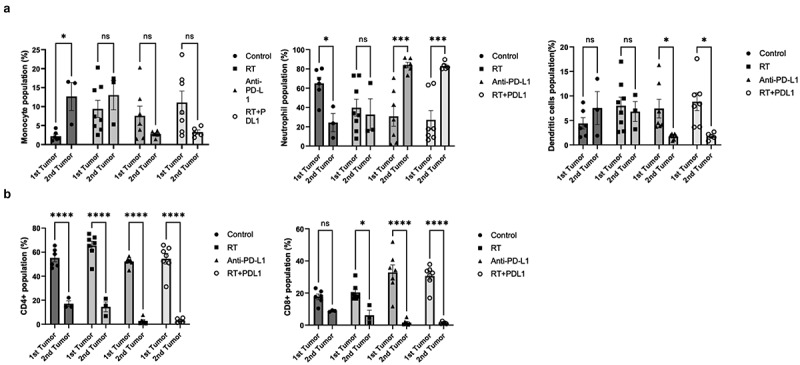
Combinatorial treatments affect the TME of re-challenge tumors differently than that of the primary tumor. A. Frequencies of monocytes, neutrophils, and dendritic cells in the primary tumors and in the re-challenge tumors (gated on CD45^+^ cells). B. Comparison of the T cell frequencies in the first tumors and the re-challenge tumors(gated on CD45^+^CD^+^). *= *p* < 0.05; **= *p* < 0.01; ***= *p* < 0.01.

Despite the apparent lack of memory establishment in the UPPL TME, we wanted to compare the immune infiltrates of the TME from the primary and the re-challenged tumors, to assess whether RT and ICI may have long-lasting effects on immune recruitment. We assessed the cellular immune landscape in tumors of re-challenged mice by flow cytometry and found that, in the groups that received anti-PD-L1 treatments, there was a significant increase in neutrophils frequencies concurrently with a decrease in dendritic cells frequencies (Figure 3a). Frequencies of monocytes within the re-challenged TME did not significantly vary when compared to the primary tumors (Figure 3a). Surprisingly, the rechallenged tumors had a significantly lower population of both CD4^+^ and CD8^+^ T cells in all treatment arms (Figure 3b), suggesting impaired local T cell recruitment in the re-challenged tumors regardless of previous treatment.

## Discussion

RT has been used as an alternative treatment for invasive bladder cancer. Although it can be immunostimulatory, it can also be immunosuppressive due to the upregulation of PD-L1. Supplementing RT with antibodies targeting PD-L1 is believed to counteract this immunosuppressive effect. We have previously demonstrated that combining RT with ICI enhances the effects of radiation in MB49 bladder tumors, simulating a basal tumor model.^24^ Little is known about the effects of radiation or combined therapy on luminal bladder tumors and in MIBC, even though luminal tumors represent in total 47% of all MIBC cases (24% luminal papillary, 15% luminal unstable, and 8% luminal nonspecific^6^). Different molecular subtypes of bladder cancer may have different markers and different TME with different immune infiltrations.^6,27^ Indeed, molecular subtyping relies on a consensus transcriptomic profile, and recent studies have further expanded that definition by relating molecular subtype, immune signature, and response to therapies.^31^ Combes, *et al*. have defined pan-cancer immune archetypes that have yet to be characterized in bladder cancer.^31^ Yet, we do know that, in bladder cancer, basal subtypes are more infiltrated by M1 macrophages, NK cells, memory CD4 and neutrophils, while luminal subtypes elicit the recruitment of activated DCs, Tregs, memory, and naïve B cells.^32^ In humans, TCGA data analysis of BCR and TCR sequencing suggests basal/squamous (Ba/sq) tumors and stroma-rich tumors infiltrating lymphocytes are more clonally expanded than in the luminal subtype.^33^ Nevertheless, retrospective analysis of the ImVigor210 trial also highlights that overall high immune infiltration (based on an immune gene score) in both ba/sq and luminal MIBC tumors correlate with the lowest response rates to immunotherapy.^34^ This hints that while efficient T cell responses and recall responses are required for potent anti-tumor immunity, factors intrinsic to the TME of MIBC luminal tumors prevent the expansion of functionally adequate T cells.

There are many factors which can impact the development of T cell activation and immune memory: lack of tumor antigens, lack of APCs recruitment, absence of T cell activation and impaired trafficking and infiltration into the tumors.^17^ In the case of a cold tumor, the low presence of antigens and initial T cell presence makes the development of immune memory less optimal. Turning a cold tumor into a hot tumor would thus require high amounts of tumor-specific antigens and high T cell presence, which might boost ICI response.^17,29^ The UPPL cell line has been demonstrated to recapitulate the bladder cancer luminal molecular subtype.^10,28^ In our studies, UPPL tumor responded to the radiation treatment with significant tumor growth delay, consistent with previous data from other studies wherein luminal molecular subtype cell lines also had similar responses to RT.^9^ However, anti-PD-L1 treatment was not able to synergize with radiation to further delay tumor growth. This lack of combined response may be attributed to the low PD-L1 expression of UPPL cells. Conversely, we found high frequencies of PD-L1^+^ cells in the immune compartment, where anti-PD-L1 treatment could additionally target those cells. However, it is likely that the anti-PD-L1 treatments were not able to boost the RT efficacy due to baseline low immune infiltration of T cells. This observation of low immune infiltration and PD-L1 expression of UPPL tumor compared to previously characterized basal-like models recapitulates luminal bladder cancer tumor features in patients.^27^ In the context of cold tumor environment where there is a lack of antigen at the start, it may be possible to use techniques such as single-cell analysis, to not only personalize treatments with chimeric antigen receptor T (CAR-T) but also identify tumor-specific antigens (TSAs).^35^ Characterizing TSAs, in terms of their abundance and heterogeneity across different cancer subtypes can enhance the understanding on how to target the tumor and augment the recognition.

There is only sparse literature on immune memory in response to cold tumor and immunotherapy, especially in the bladder cancer context. In patients with Merkel cell carcinoma, response to PD-1/PD-L1 inhibition is associated with predominant T_CM_ with high TCR editing levels within the TME.^36^ Results from experimental models of colorectal cancer using the MC38 cell line show that while RT does damage T cells, TGF-β in the TME promotes T cell radioresistance and supports T cell reprogramming toward tumor-resident-like T cells.^37^ The latter are highly radio-resistant can convert to resident memory T cells (T_RM_) and support inflammatory cell activation. Importantly, an increase in inflammatory signals is likely to drive Treg recruitment to the tumor, which may overshadow the overall response at the time point we investigated in the present study. Human data from the ImVigor210 trial supports those experimental results, with high T_RM_ infiltration, specifically CD103^+^CD8^+^ T cells correlating with better outcomes to ICI.^38^ T_RM_ infiltration in the tumor also correlated with overall increases in pro-inflammatory cytokine release.^38^ In luminal-like breast cancer treatment-naïve tumors, CD8^+^ T_RM_ is also associated with clinical benefits.^39^ Our study is restricted to the classical memory T cell population and may thus benefit from additional in-depth profiling of tumor T cells in the UPPL model, to assess the leveraging power of our combinatorial approach. Experimental models of lung and prostate cancers indeed show that radiation increases T_EM_ in the 2 weeks following treatments concurrent with transient local T_CM_ induction in the TME,^40^ while combination with ICI in lung cancer yields increased T_CM_ expansion in the spleen but not in local infiltrating T cell pools.^38^ Immunotherapy alone or combined with RT consistently led to increases in T_EM_ in the TIL compartment.^38^ Hence, there is likely a fine-tuning that needs to be performed in terms of treatment regimen kinetics: if RT stimulates neoantigen release and presentation, as well as resident T cell conversion to support better local recall responses, a treatment strategy wherein RT is given as neoadjuvant to ICI may be beneficial for T_EM_ and T_CM_ establishment. In metastatic MIBC, a retrospective analysis showed that patients receiving RT to the primary tumor prior to immunotherapy and then receiving pembrolizumab (anti-PD-1) had significantly higher objective response rates to the latter.^41^

Following treatments, we excised UPPL tumors and re-challenged mice to evaluate the effect of RT and ICI on long-lasting immunity. Re-challenge experiments showed a remarkable absence of treatment-induced immunological memory as there were no growth delays seen between the different groups when compared with primary tumors. This is associated with lower T cell frequencies within the TME of re-challenged tumors relative to primary tumors, regardless of the initial treatment arm. While not covering MIBC, evidence in patients with intermediate or high-risk NMIBC treated with BCG intravesical instillations suggests an influential role for innate memory in durable response to immunotherapy.^42^ Innate memory (also termed trained immunity) is acquired in circulating monocytes through repeated antigen exposure, which leads to epigenetic modifications, allowing for the expression of pro-inflammatory genetic programs that rely on IL-12 and IFNg and IL-6 release.^43^ Because T cell memory seemed unaffected by either RT or ICI treatments and even seemed worse following re-challenge, evaluating innate immunity and its contribution to radiation-induced anti-tumor immunity may be warranted in the luminal setting in MIBC. This is especially relevant given the high numbers of monocytes, dendritic cells, and neutrophils that were found to infiltrate UPPL in both primary tumors and re-challenged tumors. In support of this, comparing immune infiltration in the primary tumor and the re-challenge tumor, we observed high neutrophils and low T cell infiltration, especially CD8^+^ T cells, in the former. This profile is associated with worse overall survival.^30^ Other than cell analysis, it may also be important to profile the cytokine and chemokine secretion from the tumor and the immune cells to understand their interactions and activity. Studying the proteomics of the tumors can aid in identifying targets for treatments. Canale et al. have demonstrated by performing mass spectrometry on liver tumors that the tumor-infiltrating CD8 T cells upregulate AFAP1L2 in response to chronic stimulation, and the deletion of AFAP1L2 in CD8 T cells enhanced their anti-tumor activity.^44^ Another group had also characterized the two main subtypes of solid cancer (small squamous carcinoma and adenocarcinoma) and found distinct proteomes that provide insights on the different tumor development and therapeutic strategies.^45^ Groeneveld et al. studied the proteomics of 63 NMIBC and MIBC tumors and were able to find similar classification with the transcriptomics subtypes, but also found heterogeneity within the subtypes.^46^ All and all, the combination of proteomics, genomics, and TME characterizations would be important factors to consider together in targeted therapy.

In brief, we used here UPPL as a model to represent the luminal bladder cancer molecular subtype. We characterized the TME following treatments and investigated the effect of radiation and anti-PD-L1 on immunological memory. The combination treatment was not able to synergize to impact the UPPL tumor growth nor to induce effective immune memory to control tumor growth upon re-challenge. Furthermore, the conversion of a cold tumor into a hot tumor was not achieved by combinational treatments. Further studies are warranted to evaluate if other combinations of treatments could significantly increase select immune cell infiltration and activation that may help enhance radiation efficacy in an immune-cold luminal-like bladder tumor model.

## Limitations

While this study examined the global effects of immune memory development following ICI and radiation and the immune population in the TME, it did not assess the killing potential and activity of the immune cells. Recruitment of immune cells does not always indicate that the cells are active and not exhausted. We used PD-1/PD-L1 to assess immune exhaustion, but other surface markers could also indicate exhaustion. Additionally, various markers can be used to evaluate the activation status of immune cells within the TME. Moreover, the mice received UPPL cells in their flanks and not in the orthotopic bladder. The bladder may have a different environment than the flank, and there is a possibility that the treatments might have had a different effect due to the different sites.

## Methods

### Cell lines and cell culture

UPPL1540 (UPPL) syngeneic bladder cancer cell line was gifted by Dr. William Kim (University of North Carolina, Chapel Hill, NC, USA). The cells were cultured at 37° in Dulbecco’s Modified Eagle’s Medium (DMEM, Wisent, St-Bruno, QC, Canada) supplemented with 10% fetal bovine serum (FBS, Wisent) and passaged or used when reaching 80% confluency. Trypsin (Wisent) was used to detach the cells at each passage. For all experiments, the cells were passaged more than twice and no more than 10 times before use *in vivo*. Cell count was performed using Vi-cell-XR cell viability analyzer (Beckman Coulter, Mississauga, ON, Canada).

### Establishment of the syngeneic bladder cancer mouse model

As bladder cancer is more prevalent in men, C57BL/6 7–10 weeks old male mice were purchased from Charles River Laboratories, Inc. All animal experiments were done according to the Animal Ethical Care Protocol approved by our animal facility and followed the standard operating procedures (SOPs) put in place. Five million UPPL cells were injected in the right flank of the mice. The animals were monitored regularly, every 2 days following the establishment of the tumor (0.1 cm^3^). The masses were measured using an electrical caliper, and the volume was estimated using an ellipsoidal approximation formula (estimated tumor volume = 4/3 * (3.14159) * (Length/2) * (Width/2)2). Following the establishment of the tumor, the mice were randomized into the different treatment groups (*n* = 6–8) and treated accordingly: Control, RT, Anti-PD-L1, and RT + Anti-PD-L. The primary endpoint was assessed using tumor volume larger than 1.5 cm^3^ or skin ulceration. Mice that sustained severe injuries due to other causes, such as fighting, were excluded from the study.

### Radiotherapy of tumors

X-RAD SmART Irradiator Pxi 225c× (Precision X-Ray, North-Branford, CT, USA) was used to administer radiotherapy. Fluoroscopic guidance was used to image and target only the tumor before treatment started. Two doses of 5 Gy were given to the radiation treatment group mice 24 hours apart as per previous experiments from our group where the radiation strategy slowed the tumor growth in mice alone or in combination with other treatments.^25,47,48^

### Treatment with immune checkpoint inhibitor in vivo

PD-1/PD-L1 blockade treatment was administered as 200 µL intraperitoneal injections of 250 µg of InVivoMAb anti-mouse PD-L1 clone 10F:9G2 (BioxCell, Lebanon, New Hampshire, USA) in PBS (Wisent, St-Bruno, QC, Canada). The treatment was administered every other day for a total of 4 doses. Immune checkpoint treatment and radiotherapy start on the same day for the combination treatment group.

### Tissue dissociation and preparation of tumor microenvironment

The excised tumors from mice were weighed before a flow cytometry procedure was performed. The tumors for flow cytometry were collected in Roswell Park Memorial Institute 1640 (RPMI, (Wisent)) supplemented with 10% FBS. The tissues were digested using a mouse tumor dissociation kit (Miltenyi Biotec, Somerville, MA, USA) and followed by physical dissociation using a gentleMACS dissociator (Miltenyi Biotec). The samples were passed through a 70 µm cell strainer to obtain a single-cell suspension. The collected cells were treated using ACK lysing buffer (Thermofisher, Waltham, MA, USA) to lyse the red blood cells. The cells are then counted before flow cytometry analysis. If necessary, the cells are kept in MACS tissue storage solution (Miltenyi Biotec) to be used the following day. The spleens were collected in RPMI supplemented with 10% FBSs and then dissociated with a blade before being passed through a 70 µm cell strainer. The cells were treated with ACK lysing buffer and resuspended into a single-cell suspension and counted.

### Multi-parametric flow cytometric analysis

The single-cell suspensions were first stained with a viability dye (viability eFluor780, eBiosciences) followed by Fc Block (CD16/CD32 (Invitrogen, Waltham, MA, USA)). For the T cells panel, the following antibodies were used: CD45 – FITC (Biolegend, San Diego, CA, USA), CD3 – BUV737 (BD Biosciences, Franklin Lakes, NJ, USA), CD4 – AF700 (Thermofisher), CD8 – BV510 (BD Biosciences), CD44 – PECy7 (Biolegend), CD62L – PerCP/Cy5.5 (Biolegend), KLRG1– BUV395 (BD Biosciences), PD-1 – BV421 (Biolegend), TIM3 – PE (Biolegend). For the myeloid panel, the following antibodies were used: CD45 – FITC (Biolegend), CD11c – BUV737 (BD Biosciences), Ly6C – PECy7 (Biolegend), MHCII – PerCP/Cy5.5 (Biolegend), CD11b – APC (Biolegend), F4/80 – BUV395 (BD Biosciences), Ly6G – BV421 (Biolegend), PD-L1 – PE (Biolegend). Necessary fluorescence minus one (FMO) control was used for gating. The stained cells were acquired using BD LSRFortessa X-20 (BD biosciences) and analyzed using Flowjo V10. Appropriate gating strategies were applied (Suppl. Figure S1).

### Statistical analyses

Repetitive measures Kruskal-Wallis model with Dunn’s correction were used to compare flow cytometry cell frequencies and tumor volume means across treatment groups and/or response groups. Kaplan – Meier curves were used to compare differences in survival and tested for significance with log-rank tests. Statistical comparisons between treatment groups were tested using Mann – Whitney U tests. A two-sided *p* value < .05 was considered statistically significant.

## Supplementary Material

Supplemental Material

## Data Availability

The data that support the findings of this study are available from the corresponding author, WK, upon reasonable request.
