# Supplementary figures and images for: Effects of combined radiotherapy with immune checkpoint blockade on immunological memory in luminal-like subtype murine bladder cancer model
Source: Cancer Biol Ther. 2024 Jun 11;25(1):2365452. doi: 10.1080/15384047.2024.2365452 (PMC11174127; doi:10.1080/15384047.2024.2365452)

**Supplemental Figure**


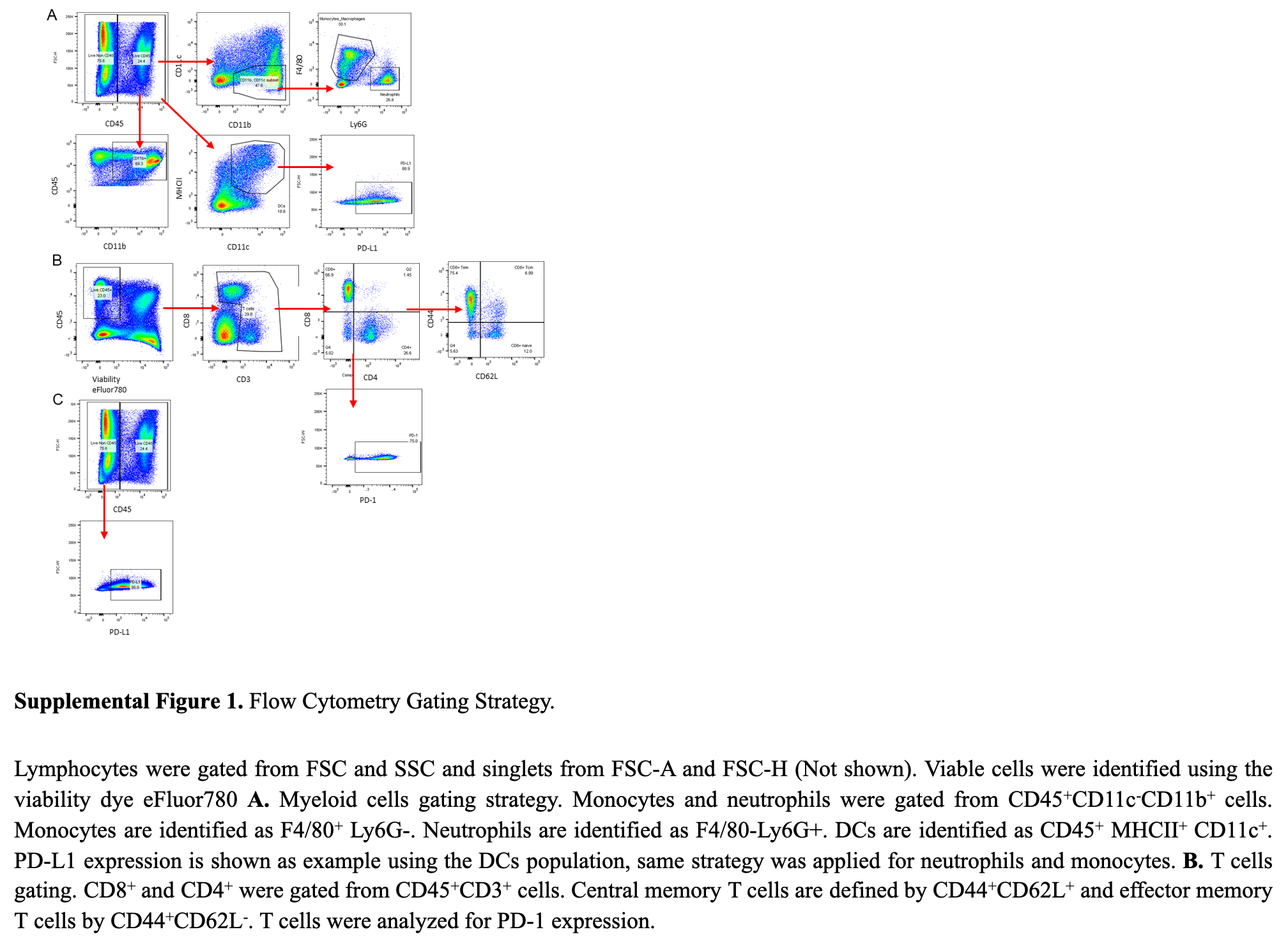

Supplement: Supplemental Material [file KCBT_A_2365452_SM7858.docx]
